# Adherence to 24-Hour Movement Guidelines for the Early Years and associations with social-cognitive development among Australian preschool children

**DOI:** 10.1186/s12889-017-4858-7

**Published:** 2017-11-20

**Authors:** Dylan P. Cliff, Jade McNeill, Stewart A. Vella, Steven J. Howard, Rute Santos, Marijka Batterham, Edward Melhuish, Anthony D. Okely, Marc de Rosnay

**Affiliations:** 1 Early Start, Faculty of Social Sciences, University of Wollongong, Northfields Ave, Wollongong, NSW 2522 Australia; 2 Illawarra Health and Medical Research Institute, University of Wollongong, Wollongong, Australia; 3 Research Centre in Physical Activity, Health and Leisure, Faculty of Sports, University of Porto, Porto, Portugal; 4 Statistical Consulting Service, School of Mathematics and Applied Statistics, Faculty of Engineering and Information Sciences, University of Wollongong, Wollongong, Australia; 5 Department of Education, University of Oxford, Oxford, UK; 6 Psychological Sciences, Birkbeck, University of London, London, UK

**Keywords:** Early childhood, Physical activity, Active play, Screen time, Electronic media, Sleep, Theory of mind, Emotion understanding, Health behaviour

## Abstract

**Background:**

The new Australian 24-Hour Movement Guidelines for the Early Years recommend that, for preschoolers, a healthy 24-h includes: i) ≥180 min of physical activity, including ≥60 min of energetic play, ii) ≤1 h of sedentary screen time, and iii) 10–13 h of good quality sleep. Using an Australian sample, this study reports the proportion of preschool children meeting these guidelines and investigates associations with social-cognitive development.

**Methods:**

Data from 248 preschool children (mean age = 4.2 ± 0.6 years, 57% boys) participating in the PATH-ABC study were analyzed. Children completed direct assessments of physical activity (accelerometry) and social cognition (the Test of Emotional Comprehension (TEC) and Theory of Mind (ToM)). Parents reported on children’s screen time and sleep. Children were categorised as meeting/not meeting: i) individual guidelines, ii) combinations of two guidelines, or iii) all three guidelines. Associations were examined using linear regression adjusting for child age, sex, vocabulary, area level socio-economic status and childcare level clustering.

**Results:**

High proportions of children met the physical activity (93.1%) and sleep (88.7%) guidelines, whereas fewer met the screen time guideline (17.3%). Overall, 14.9% of children met all three guidelines. Children meeting the sleep guideline performed better on TEC than those who did not (mean difference [MD] = 1.41; 95% confidence interval (CI) = 0.36, 2.47). Children meeting the sleep and physical activity or sleep and screen time guidelines also performed better on TEC (MD = 1.36; 95% CI = 0.31, 2.41) and ToM (MD = 0.25; 95% CI = −0.002, 0.50; *p* = 0.05), respectively, than those who did not. Meeting all three guidelines was associated with better ToM performance (MD = 0.28; 95% CI = −0.002, 0.48, *p* = 0.05), while meeting a larger number of guidelines was associated with better TEC (3 or 2 vs. 1/none, *p* < 0.02) and ToM performance (3 vs. 2, *p* = 0.03).

**Conclusions:**

Strategies to promote adherence to the 24-Hour Movement Behaviour Guidelines for the Early Years among preschool children are warranted. Supporting preschool children to meet all guidelines or more guidelines, particularly the sleep and screen time guidelines, may be beneficial for their social-cognitive development.

## Background

Systematic reviews indicate that physical activity [1], recreational screen time [2] and sleep [3] are individually associated with health and developmental outcomes among young children. Recently, a shift has occurred from focusing on the potential benefits or harms of engaging in these behaviours individually, to the more holistic conceptualization that children’s health and development is likely to be optimized if adequate levels of key health behaviours can be achieved simultaneously [4] such as sufficient physical activity and sleep, and limited exposure to electronic screen devices for recreation. Studies [5, 6] and reviews [7–9] in school-aged children and adolescents support this approach and, as a result, Canada released the first 24-h Movement Guidelines for Children and Youth in 2016 that integrated recommendations for physical activity, sedentary behaviour, and sleep [10]. The Canadian 24-Hour Movement Guidelines for the Early Years [11] and the concurrent adolopment of these guidelines in Australia [12], published in this special issue of *BMC Public Health,* also promote the integration of physical activity, sedentary behaviour, and sleep for health benefits among young children. These guidelines recommend that, for preschoolers, a healthy 24 h includes: i) at least 180 min of physical activity, of which at least 60 min is energetic play, ii) no more than 1 h of sedentary screen time, and iii) 10 to 13 h of good quality sleep.

Relative to school-aged children, however, the evidence supporting the integration of these health behaviours for health and developmental benefits in young children is still emerging, and the small number of existing studies have investigated associations for a limited range of outcomes such as adiposity, fitness, growth and motor development [13, 14]. The systematic review in this special issue conducted by Kuzik and colleagues [13] of studies examining the combined effects of these movement behaviours on health indicators in young children indicates that no studies have examined the potential benefits for other important areas, such as cognitive or social development.

Social cognition, which includes children’s understanding of others’ minds and emotions and the application of this information, has been shown to be an important predictor of early social behaviour and integration [15–17] that is highly sensitive to the child’s social and communicative environment [18]. Physical activity, which often involves group-based participation, and screen time, which removes young children from interpersonal communications, might exert indirect effects on children’s social behaviour (e.g., behaviour problems, social skills, prosocial behaviour, etc.) through their potential influences on the development of social-cognitive capacities, which have been shown to change rapidly in the preschool period [19]. Likewise children’s social-cognitive capacities rely heavily on higher-order cognitive processes called executive functions [20], and there is some evidence that sleep [21], screen behaviours [22] and physical activity [23] might be associated with executive functions in young children. In keeping with this interpretation, there is some cross-sectional research which suggests that young children’s television viewing behaviours [24, 25] and sleep duration [25] may be detrimentally associated with social cognitive development.

With the release of the new 24-Hour Movement Guidelines for the Early Years, there is a need to understand the proportion of young children meeting the guidelines, and if adherence is associated with health and developmental outcomes for children. Furthermore, examining associations of different combinations of behaviours adds to the currently limited evidence base on how the behaviours interact to influence health and development in early childhood. Therefore, the purpose of this study was to examine: i) adherence to the individual and integrated 24-Hour Movement Guidelines for the Early Years, and ii) associations between guideline adherence and social-cognitive development in Australian preschool children.

## Methods

### Study design

The cross-sectional analyses in this paper draw on data from the Preschool Activity, Technology, Health, Adiposity, Behaviour and Cognition (PATH-ABC) observational study, for which the methods have been described [26].

### Setting, sampling and recruitment

Participating children were recruited from Early Childhood Education and Care (ECEC) centres in the Illawarra region of New South Wales, Australia between April and December 2015. Centres were invited to participate based on a stratified sampling process. Firstly, all ECEC centres in the region were categorised into low (deciles 1–4), medium (deciles 5–7) or high (deciles 8–10) socio-economic areas based on their suburb and the 2011 Socio-Economic Indices For Areas (SEIFA) Index of Relative Socio-economic Advantage and Disadvantage (IRSAD) [27]. The number of ECEC centres invited to participate from each socio-economic group was proportional to the number within the population of centres. Data collection occurred from April to December 2015 in children’s ECEC centres, including preschools and day-care centres, by 2–4 trained assessors over 1–5 days.

### Participants

Children were eligible to participate in the study if they attended an ECEC centre and were ≥3 years-of-age (children attending ECEC centres in Australia are generally <6 years of age, as was the case for the study sample), generally healthy, and experiencing typical development. Children were ineligible if they had a learning or physical disability, known motor delay, or a diagnosed medical or psychological condition (e.g., conduct disorder) that would affect the results of the study.

### Study protocol

Prior to participation in the study, Directors of participating ECEC centres provided electronic or paper versions of information sheets and consent forms to all eligible children’s parents/carers. Following informed consent, parents/carers provided demographic information and other data via surveys. After verbal consent, trained data collectors completed assessments with children in a quiet area of the ECEC centre, away from the main group of children but within the supervision of the educators.

### Measures

#### Physical activity

Physical activity was measured objectively using accelerometers (ActiGraph GT3X+). The ActiGraph has established acceptability, validity and reliability in preschool children [28, 29]. Children wore the accelerometer around their waist on an elastic belt at the right hip, anterior to the iliac crest, 24-h/day for one week. Non-wear time was defined as ≥20 min of consecutive “0” counts, and children’s physical activity data were included in analyses if they had a least 1 day of valid data (≥360 min of valid wear time [30]) between 5 am to 11 pm. Valid weekend day data were not a requirement for inclusion because evidence in preschoolers indicates that reliability of physical activity estimates is not substantially increased by inclusion of a weekend day [30]. Total physical activity (TPA; ≥25c/15 s) (i.e., light-, moderate- or vigorous-intensity physical activity) and moderate- to vigorous-intensity physical activity (MVPA; ≥420c/15 s) were defined using age-appropriate cut-points that have been shown to be most accurate in young children [28, 31, 32]. Children averaged 740.6 ± 120.9 min/day of valid wear time over 6.2 ± 2.4 days, and 223 (90%) of the sample had ≥3 valid days, while 9 (3.6%) had only 1 valid day (which in all cases were weekdays).

#### Screen-based entertainment

Parents reported on the time children usually spent in screen-based behaviours during a typical week [33]. This included the total amount of time their child spent in separate screen-based behaviours on weekdays (Monday to Friday) and on weekends (Saturday and Sunday). Original items were modified to include mobile devices as follows: television programs/movies/internet clips on traditional devices (e.g., TV/DVD), television programs/movies/internet clips on other devices (e.g., tablet, DVD in car, computer, laptop, mobile phone), games/apps on portable handheld devices (e.g., tablet, mobile phone, handheld game system), console games (non-active) (e.g., PlayStation, Xbox), and console games (active) (e.g., Wii, Xbox Kinect). Weekday and weekend day minutes were summed across the week to calculate total weekly time spent in each behaviour, excluding console games (active). Subsequently, weekly totals for each behaviour were summed and averaged to derive the child’s average daily time spent in screen-based entertainment.

#### Sleep

Parents reported children’s usual daily sleep duration. This approach has been validated against estimates from sleep logs and objective actigraphy in young children [34].

#### Emotional understanding

Emotional understanding was examined because of the role that early emotional competence plays in children’s social development [35] and academic achievement [36], and particularly as a potential foundational component of children’s social cognitive development; their understanding that people have different desires, thoughts and beliefs that may influence their behaviours [37]. Four components of the Test of Emotion Comprehension (TEC) test battery were administered to assess participants’ emotional understanding, namely: *recognition* (I), *external cause* (II), *desire* (III), and *belief* (IV; [38]. This assessment was chosen because scalogram analyses reported by Pons et al. [38] indicate that the scale is valid and reliable in preschool children. Specifically, using this scale, it has been demonstrated that: i) children display a clear improvement with age on each of nine key components of emotional development, ii) three developmental phases can be identified, each characterised by the emergence of three of the nine components, ii) associations exist among components within a given phase, and iv) hierarchical associations exist among components from successive phases [38]. Corresponding with participating children’s developmental phase, tasks related to the first four emotional components were administered.

For the first TEC component, recognition, children were asked to point to emotional expressions that corresponded to five different emotion labels (i.e., *happy*, *sad*, *angry*, *scared* and *just okay*). This first item establishes whether children understand the correspondence between conventional emotion labels and canonical, simplified expressions. In subsequent TEC components, children’s responses are given by pointing at a selection (2 × 2) of emotion expressions (e.g., happy, sad, angry, just alright) following a story scenario that has an emotional outcome or consequence for the protagonist. Thus, children’s responses are closed and non-verbal. For example, in component II, external causes, children are shown a picture, read an accompanying story, and are asked to select an emotional outcome (presented as four emotion faces) that would be provoked by an external cause (e.g., a Birthday party) displayed in each of five separate vignettes. Details for components III and IV are provided in Pons et al. [38]. The TEC can be scored for each item (components I-IV have 23 items) and Total TEC scores were used in analyses.

#### Theory of mind

A scaled set of five age-appropriate tasks from the global assessment used by Wellman and Liu [19] were used to assess Theory of Mind (ToM). These tests assess how a child is able to attribute core mental states to others; specifically, desires, knowledge, true beliefs, false beliefs, and concealed emotions. These tasks were chosen because: i) psychometric testing indicates that they are valid and scalable in preschoolers [19], ii) they are comprehensive, spanning a wide range of tasks and ages [19], and iii) they are widely used internationally and are the current bench mark by which ToM development is assessed in the preschool period [39]. Each task is administered in a specific order due to the demonstrated progression of a child’s development. All tasks require the participants to answer one target question depicted in a story book about a protagonist’s mental state or behaviour and one control or contrast question about reality or someone else’s mental state or behaviour. These tasks can be completed in approximately 5 min per child and children are awarded a pass (1)/fail (0) response for each of the five items. Total ToM scores out of five were used in analyses.

### Data reduction and analysis

As recommended for surveillance studies, preschoolers were classified as meeting the overall *24-Hour Movement Guidelines for the Early Years* if they met physical activity (≥ 180 min/day of TPA including ≥60 min/day of MVPA), screen time (≤ 1 h/day), and sleep duration (10–13 h/day) recommendations [11]. Children were categorised as meeting or not meeting: i) individual guidelines, ii) combinations of any two guidelines, or iii) all three guidelines. Associations were examined for i) individual guidelines, ii) combinations of any two guidelines, or iii) the number of guidelines met, and iv) the overall integrated guidelines using linear regression models adjusting for child age, sex, expressive vocabulary [40], suburb-level socio-economic status (Socio-Economic Indices For Areas (SEIFA) Index of Relative Socio-economic Advantage and Disadvantage (IRSAD) [27] and the clustered sampling approach. Because early vocabulary is intimately associated with developmental outcomes in childhood [41], expressive vocabulary was included as a covariate. This was assessed using the valid and reliable Early Years Toolbox Vocabulary task [40]. This 45-item measure of a child’s expressive vocabulary development requires children to verbally produce the correct label for each depicted stimulus. Participants respond verbally and a data collector recorded this response. In cases of an incorrect label initially being produced, the data collector prompted participants by asking ‘what else might this be called’ until either a correct production or some indication that the child was unable to produce the required word. Statistical analyses were conducted in Stata v.13 (Stata Corporation, College Station, TX).

## Results

Of the 490 children recruited to the PATH-ABC study, 161 were missing parent-reported screen time data, 34 were missing ToM data, 21 were missing physical activity data, 13 were missing vocabulary data, 9 were missing parent-reported sleep data, 4 were missing suburb-level socio-economic status data, and 5 additional children were missing TEC data. Therefore, 248 and 243 participants were included in analyses for ToM and TEC, respectively. Children with missing data had slightly but significantly lower suburb-level socio-economic status (mean difference = 13.4; 95% confidence interval (CI) = 1.9, 24.8 units) and vocabulary scores (mean difference = 2.1; 95% CI = 0.7, 3.4 units) than those in the analytic sample.

Participant descriptive data are reported in Table 1. Participants averaged more than 6 h/day in TPA, of which 1.7 h/day was MVPA, 2.3 h/day of screen time and ~10.5 h/day of sleep. Girls were younger than boys and spent less time in MVPA (*p <* 0.05), however, there were no other statistically significant differences in behaviours, covariates or outcomes.

**Table 1:** Participant descriptive characteristics and differences by sex

	Total Sample (*n* = 248)	Boys (*n* = 141)	Girls (*n* = 107)	*p*
Age (M, SD)	4.2 ± 0.6	4.4 ± 0.7	4.0 ± 0.6	<0.001
SES (IRSAD) (M, SD)	1020 ± 61	1025 ± 62	1013 ± 58	0.13
Vocabulary score (M, SD)	27.2 ± 6.5	27.6 ± 6.5	26.6 ± 6.5	0.23
Movement behaviours				
Physical activity – TPA (min/d) (M, SD)	373.3 ± 71.3^#^	376.7 ± 70.5	368.8 ± 72.3	0.39
Physical activity – MVPA (min/d) (M, SD)	102.5 ± 33.9^#^	108.9 ± 36.0	94.1 ± 29.0	<0.001
Screen time (min/d) (M, SD)	139.8 ± 83.4	144.3 ± 86.0	134.0 ± 79.8	0.34
Sleep (hrs/d) (M, SD)	10.5 ± 1.0	10.5 ± 1.0	10.5 ± 1.0	0.81
Social Cognition				
TEC* (M, SD)	9.56 ± 2.87	9.78 ± 2.84	9.25 ± 2.89	0.17
ToM (M, SD)	2.30 ± 1.08	2.33 ± 1.06	2.23 ± 1.08	0.56

*IRSAD* Index of relative socio-economic advantage and disadvantage, *M* mean, *MVPA* moderate- to vigorous-intensity physical activity, *SD* standard deviation, *SES* socio-economic status, *TEC* Test of Emotion Comprehension, *ToM* Theory of Mind, *TPA* total physical activity

*n = 243; boys = 139, girls = 104. ^#^Out of 740.6 ± 120.9 min/day of valid wear time

With respect to the proportion of children meeting each individual movement behaviour guideline, high proportions of children met the physical activity (93.1%) and sleep (88.7%) guidelines, whereas fewer met the screen time guideline (17.3%) (Table 2). Consequently, high proportions of children met the physical activity and sleep guidelines combined (82.7%), but fewer met the physical activity and screen time guidelines (16.9%), or the sleep and screen time guidelines (16.5%) (Table 3). Overall, 14.9% of children met the integrated 24-h Movement Guidelines for the Early Years (i.e., met all three individual movement behaviour guidelines).

**Table 2:** Meeting individual guidelines and social-cognitive development

	Mean (95% CI) for each group		Mean (95% CI) difference between groups	
Physical Activity (≥180 min/d of TPA including ≥60 min/d of MVPA)	Meeting (*n* = 231)	Not Meeting (*n* = 17)	Meeting - Not Meeting	*p*
TEC total*	9.61 (9.06, 10.17)	8.52 (6.85, 10.20)	1.09 (−0.32, 2.50)	0.121
ToM total	2.29 (2.10, 2.49)	2.37 (1.92, 2.83)	−0.07 (−0.52, 0.37)	0.729
Screen Time (≤60 min/d)	Meeting (*n* = 43)	Not Meeting (*n* = 205)	Meeting - Not Meeting	*p*
TEC total*	10.06 (9.24, 10.87)	9.43 (8.78, 10.08)	0.63 (−0.25, 1.50)	0.150
ToM total	2.43 (2.22, 2.63)	2.27 (2.07, 2.47)	0.16 (−0.06, 0.38)	0.145
Sleep (≥10 h/d & ≤13 h/d)	Meeting (*n* = 220)	Not Meeting (*n* = 28)	Meeting - Not Meeting	*p*
TEC total*	9.70 (9.11, 10.29)	8.28 (7.14, 9.43)	1.41 (0.36, 2.47)	0.011
ToM total	2.35 (2.12, 2.58)	1.86 (1.35, 2.37)	0.50 (−0.14, 1.13)	0.119

Findings are from linear regression models adjusted for child age, sex, vocabulary, suburb-level socio-economic status & childcare-level clustering

*SES* socio-economic status, *TEC* Test of Emotion Comprehension, *ToM* Theory of Mind, *TPA* total physical activity

*n = 243

**Table 3:** The combination of guidelines met and social-cognitive development

	Mean (95% CI) for each group		Mean (95% CI) difference between groups	
Physical Activity + Screen Time	Meeting (*n* = 42)	Not Meeting (*n* = 206)	Meeting - Not Meeting	*p*
TEC total*	10.04 (9.20, 10.88)	9.44 (8.79, 10.08)	0.60 (−0.29, 1.50)	0.171
ToM total	2.45 (2.23, 2.67)	2.27 (2.07, 2.47)	0.18 (−0.06, 0.43)	0.136
Physical Activity + Sleep	Meeting (n = 205)	Not Meeting (n = 43)	Meeting - Not Meeting	*p*
TEC total*	9.78 (9.22, 10.33)	8.42 (7.23, 9.60)	1.36 (0.31, 2.41)	0.014
ToM total	2.35 (2.11, 2.58)	2.07 (1.66, 2.48)	0.28 (−0.25, 0.81)	0.278
Sleep + Screen Time	Meeting (n = 41)	Not Meeting (*n* = 207)	Meeting - Not Meeting	*p*
TEC total*	10.13 (9.31, 10.96)	9.42 (8.78, 10.06)	0.71 (−0.15, 1.57)	0.100
ToM total	2.51 (2.25, 2.77)	2.26 (2.06, 2.45)	0.25 (−0.002, 0.50)	0.052

Findings are from linear regression models adjusted for child age, sex, vocabulary, suburb-level socio-economic status & childcare-level clustering

*SES* socio-economic status, *TEC* Test of Emotion Comprehension, *ToM* Theory of Mind, *TPA* total physical activity

*n = 243

The associations between meeting each individual movement behaviour guideline and TEC and ToM are report in Table 2. Although differences were in favour of children meeting individual guidelines, meeting the physical activity or screen time guidelines was not associated with TEC or ToM performance. Children meeting the sleep guideline, however, performed significantly better on TEC than those who did not (mean difference = 1.41; 95% CI = 0.36, 2.47).

The associations between meeting combinations of movement behaviour guidelines and TEC and ToM are report in Table 3. Differences were consistently in favour of children meeting a combination of guidelines. Children meeting the physical activity and sleep guidelines performed significantly better on TEC than those who did not (mean difference = 1.36; 95% CI = 0.31, 2.41), whereas the difference in ToM performance between children meeting and not meeting the sleep and screen time guidelines approached significance (mean difference = 0.25; 95% CI = −0.002, 0.50; *p* = 0.052).

The associations between the number of guidelines met and TEC and ToM are reported in Table 4. Dose-response relationships were evident, in that meeting more guidelines was generally associated with better TEC and ToM performance. Compared to children meeting one/no guidelines, TEC performance was higher in children meeting two (mean difference = 1.37; 95% CI = 0.34, 2.39) or three (mean difference = 1.78; 95% CI = 0.27, 3.29) guidelines. Differences in ToM performance between children meeting two or three guidelines were also statistically significant (mean difference = 0.29; 95% CI = 0.03, 0.55, *p* = 0.03). Although the mean difference in ToM performance between groups was bigger between children meeting one/no guidelines and three guidelines compared to children meeting two or three guidelines, the smaller sample sizes resulted in wider confidence intervals and differences between children meeting one/no guidelines (*n* = 41) and three guidelines (*n* = 37) approached significance (mean difference = 0.47; 95% CI = −0.08, 1.02, *p* = 0.09).

**Table 4:** The number of guidelines met and social-cognitive development

	Mean (95% CI) for each group			Mean (95% CI) difference between groups			
	Meeting 0^#^ or 1 guideline (*n* = 41)	Meeting 2 guidelines (*n* = 170)	Meeting 3 guidelines (*n* = 37)	2–1	3–1	3–2	P for trend
TEC total*	8.34 (7.09, 9.58)	9.70 (9.16, 10.24)	10.11 (9.21, 11.02)	1.37 (0.34, 2.39), *p* = 0.01	1.78 (0.27, 3.29), *p* = 0.02	0.41 (−0.40, 1.22), *p* = 0.30	0.034
ToM total	2.10 (1.68, 2.52)	2.29 (2.04, 2.53)	2.57 (2.33, 2.82)	0.19 (−0.37, 0.74), *p* = 0.49	0.47 (−0.08, 1.02), *p* = 0.09	0.29 (0.03, 0.55), *p* = 0.03)	0.095

Findings are from linear regression models adjusted for child age, sex, vocabulary, suburb-level socio-economic status & childcare-level clustering

*TEC* Test of Emotion Comprehension, *ToM* Theory of Mind

^#^ *n* = 2 for 0 guidelines met; *n = 243

The associations between meeting the 24-h movement guidelines and TEC and ToM are reported in Table 5. Differences in ToM performance between children meeting the 24-h movement guidelines and those who did not approached statistical significance (mean difference = 0.28; 95% CI = −0.002, 0.48, *p* = 0.051). Although TEC performance was higher among children meeting the 24-h movement guidelines compared to those who did not, the difference was not statistically significant (mean difference = 0.69; 95% CI = −0.19, 1.57).

**Table 5:** Meeting 24 h movement guidelines (all 3 behaviours) and social-cognitive development

	Mean (95% CI) for each group		Mean (95% CI) difference between groups	
	Meeting guidelines (*n* = 37)	Not meeting guidelines (*n* = 211)	Meeting guidelines - Not meeting guidelines	*p*
TEC total*	10.12 (9.26, 11.00)	9.43 (8.79, 10.06)	0.69 (−0.19, 1.57)	0.116
ToM total	2.53 (2.25, 2.81)	2.25 (2.06, 2.45)	0.28 (−0.002, 0.48)	0.051

Findings are from linear regression models adjusted for child age, sex, vocabulary, suburb-level socio-economic status & childcare-level clustering

*TEC* Test of Emotion Comprehension, *ToM* Theory of Mind

*n = 243

## Discussion

This study reported the proportion of preschool children meeting the new Australian 24-h Movement Guidelines for the Early Years, and examined associations between meeting guidelines and young children’s social-cognitive development. In this sample, ~90% of preschool children met the individual physical activity and sleep guidelines, whereas only ~17% met the screen time guidelines. As a result, ~15% met the overall 24-h Movement Guidelines for the Early Years. Meeting the 24-h Movement Guidelines for the Early Years was associated with better social-cognitive development. Specifically, meeting all three individual movement behaviour guidelines was marginally associated with better ToM performance, while meeting a larger number of movement behaviour guidelines was associated with better TEC and ToM performance. Meeting the sleep guideline appeared to be more strongly associated with social-cognitive development than meeting the physical activity and screen time guidelines.

Our findings in relation to the proportion of young children meeting the new Canadian and Australian 24-h Movement Guidelines for the Early Years are reasonably consistent with other papers in this special issue. For example, using nationally representative data, Chaput and colleagues [42] reported that high proportions of Canadian preschool children met the sleep (82%) guidelines, whereas fewer met the screen time guideline (25%) and the integrated guidelines (13%). Lee and colleagues [43] and Santos and colleagues [44] reported findings in Canadian and Australian toddlers, respectively, that were similar to our study, in that high proportions met the physical activity (99% and 97%, respectively) and sleep (83% and 80%, respectively) guidelines, and low proportions met the screen time (15% and 11%, respectively) and overall guidelines (12% and 9%, respectively). One difference between the studies was that the proportion of Canadian preschoolers meeting the physical activity guideline (62%) was lower than in our study, and studies in Canadian [43] and Australian toddlers [44]. The study of Canadian preschool children was the only study of the four in a representative sample, so this may have contributed to differences in the findings. However, the study of Canadian preschool children also had methodological differences related to the measurement of physical activity, as it used a different type of physical activity monitor (Actical vs. ActiGraph), with different cut-points to define physical activity, and a longer sampling frequency (60s vs. 15 s) for a portion of the sample. These factors may have contributed to the lower proportion of children meeting the physical activity guideline in that study [42], compared to our study and those in Canadian [43] and Australian toddlers [44]. Irrespective of this difference, strategies and programs to promote adherence to the guidelines among young children are warranted, particularly to reduce recreational screen time and support children in meeting the guideline of ≤1 h/day.

To our knowledge, this is the first study to demonstrate that the combination of sufficient physical activity and sleep, and limited screen time is associated with social-cognitive development in preschool children. The associations appeared to be strongest for sleep, and to a lesser extent, screen time, and other studies are consistent in supporting the association between these behaviours and young children’s social-cognitive development. For example, Nathanson and colleagues found that aspects of preschool children’s screen time, such as having a television in their bedroom [24], or night-time television viewing [25], were cross-sectionally associated with poorer ToM performance. Likewise, Nathanson and colleagues [25] also reported that shorter sleep duration was associated with poorer ToM performance in the same sample of preschool children.

There are a number of plausible mechanisms that might contribute to the associations between screen time, sleep and social-cognitive development in preschool children. For example, high levels of screen time [22] and insufficient sleep [21] might directly interfere with young children’s higher-order cognitive processes or executive functions, which are fundamental for their social-cognitive development [20]. Likewise, screen time might indirectly influence young children’s social cognition by displacing opportunities for social interaction with adults and children [45] that are critical for development in this domain [18]. Because higher levels of screen time predict shorter sleep durations during early childhood [46], it may be hypothesised that screen time might impact social cognition through decreasing sleep duration. However, the cross-sectional analysis by Nathanson et al. [25] found that sleep duration did not mediate associations between screen time and ToM performance. Although additional longitudinal and experimental studies may be required to better understand the mechanisms through which screen time and sleep might influence social cognition in young children, our findings of associations suggest that programs and strategies to support children in reducing recreational screen time and achieving sufficient sleep are warranted and may contribute to optimising their social-cognitive development.

There were limitations that may have impacted our findings that should be taken into consideration. Although the PATH-ABC study used a representative sampling approach, a considerable proportion of participants could not be included in the analytic sample for the current analyses and excluded participants had lower suburb-level socio-economic status and vocabulary scores. This may affect the generalisability of our findings, particularly in relation to the proportion of children meeting the guidelines. Likewise, although the sample contained sufficient variability in exposures and outcomes to demonstrate novel associations between meeting the guidelines and social-cognitive development, the sample sizes for some sub-groups (e.g., not meeting the physical activity guidelines) were small and hence could not demonstrate that meaningful differences in outcomes were statistically significant. Varied wear time was not accounted for in the analysis because it was perceived that the minimum daily wear time of 360 min was sufficient for children to demonstrate adherence to the physical activity guideline of ≥180 min/day of TPA including ≥60 min/day of MVPA (i.e., ≥50% of monitored waking hours in TPA or <50% in sedentary behavior), and other approaches such as using criteria based on a percentage of valid wear time might disadvantage participants who better complied with the accelerometry protocol and accumulated more wear time. Although 93% of children in our sample met the physical activity guideline, it is plausible that variations in valid wear time might influence estimates of compliance. Likewise, 10% of the sample had <3 days of valid accelerometry data and this may have affected the reliability of their physical activity assessments [30].

As reflected in the guidelines, our analyses focused only on the average daily duration of physical activity, screen time and sleep. Other aspects of these behaviours, such as sleep disturbances or quality, the quality, type, or content of screen time [47, 48], the timing of television viewing or exposure to background television, or the quality of social interactions during screen time or physical activity, may potentially influence children’s social-cognitive development, but were not included in our analyses. Furthermore, assessments of screen time and sleep may have been affected by reporting biases. Finally, our analyses are cross-sectional, and additional longitudinal and experimental investigations are required to better understand the causal nature of associations between health behaviours or guideline adherence and social-cognitive development in young children, as well as the possible influence of other factors such as parenting. It is possible that an unmeasured factor such as parenting characteristics may have also influenced both adherence to 24-h Movement Guidelines for the Early Years and children’s social cognition, thus producing an association between meeting the guidelines and social cognition that may not be causal.

In conclusion, only ~15% of our sample of preschool children met the new Australian 24-h Movement Guidelines for the Early Years, which integrate guidelines for physical activity, screen time and sleep, predominantly because only 17% of children met the screen time guideline of ≤1 h/day. Strategies to promote adherence with the guidelines, particularly the screen time guideline, are therefore warranted. Promoting adherence to the guidelines may have important developmental implications for preschool children, in that meeting the overall guidelines (i.e., for all three movement behaviours) and meeting a larger number of guidelines were associated with better social-cognitive development. Findings also indicated that supporting preschool children to meet the sleep guideline and, to a lesser extent, also the screen time guideline, might be particularly beneficial for children’s social-cognitive development.
